# miR-127/3p Inhibits Cell Migration in Lung Adenocarcinoma Under Hypoxic and Normal Oxygen Conditions

**DOI:** 10.17912/micropub.biology.001355

**Published:** 2024-10-16

**Authors:** Jackson Lipscomb, Kassidy Gray, Tuesday Melton, Parker Nelson, Alyssa Rye, Christin L. Pruett, Nathan S. Reyna

**Affiliations:** 1 Biology, Ouachita Baptist University, Arkadelphia, Arkansas, United States

## Abstract

MicroRNAs are small noncoding nucleotides that serve as intracellular and extracellular signaling molecules. A previous collaboration found miR-127/3p circulation in the blood of breast cancer patients correlated with improved patient recovery and prognosis. While this study exclusively focused on breast cancer patients, data mining of the TCGA databases indicated that miR-127/3p may be positively associated with outcomes in other cancer types. In our study, A549 lung adenocarcinoma cells were transfected with miR-127/3p using Cell Block protocols produced by the Cell Biology Education Consortium (CBEC). After transfection, cell migration (scratch/wound healing) assays were used to determine the role miR-127/3p plays in the tumor microenvironment. To mimic and test this environment, transfected cells were incubated in normal oxygen (normoxic) and low oxygen (hypoxic) environments. We found that miR-127/3p inhibited cell migration in both normal oxygen and hypoxic environments. These results help elucidate the role miR-127/3p plays in the prevention of metastasis and further highlight its potential as a positive biomarker.

**Figure 1. The Role of miR-127/3p in the Tumor Microenvironment:**
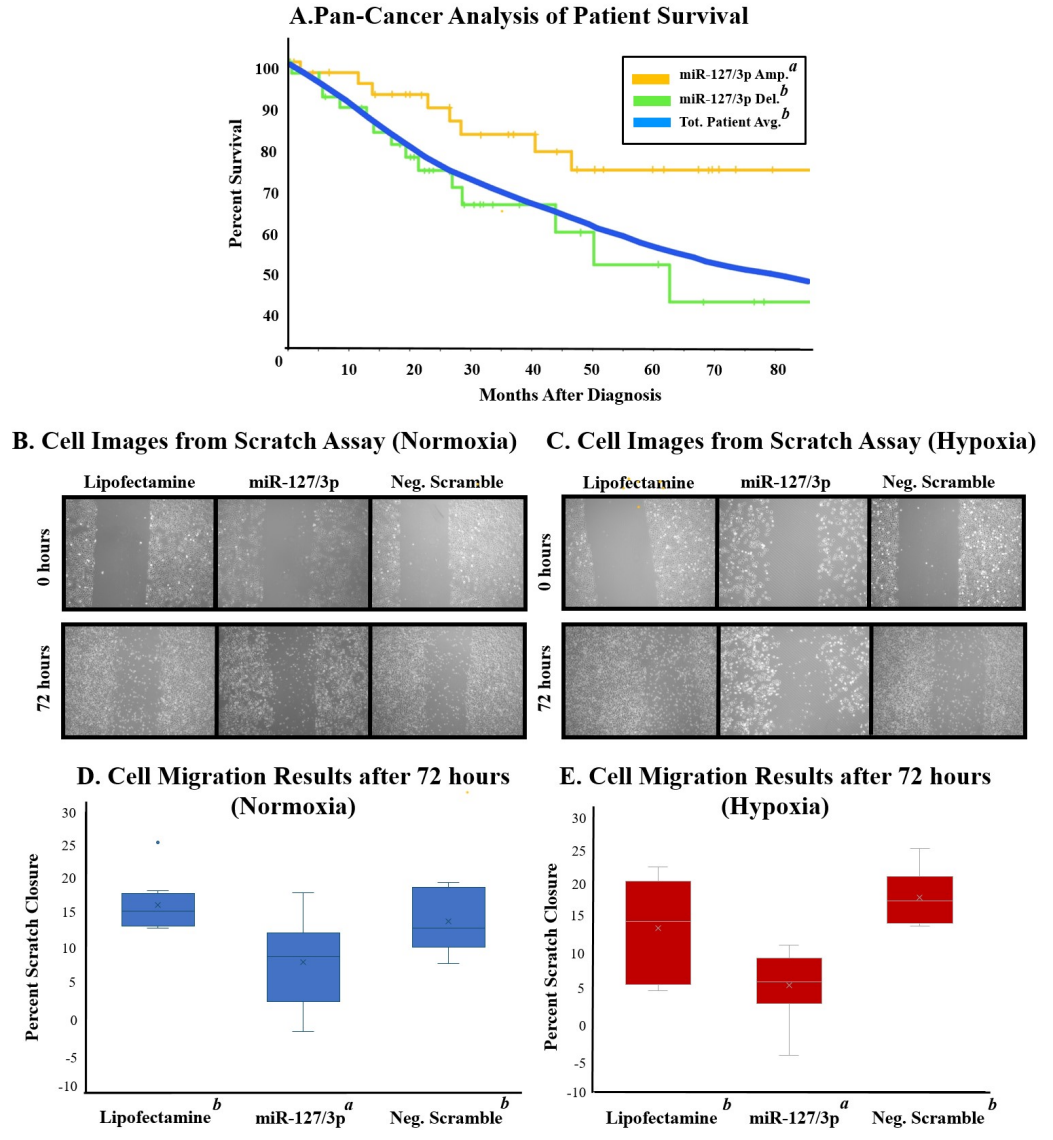
A) Pan-Cancer analysis. Average survivability of all cancer subtypes (blue) compared to miR-127/3p amplification (orange) and homologous deletion (green). Amplification was statistically different from the total patient average and homologous deletion. B) Cell images showing cell migration from 0 to 72 hours under normal oxygen conditions. C) Cell images showing cell migration from 0 to 72 hours under hypoxic conditions. Pictures at 0 hours were taken after hypoxic incubation. D) Cell migration results under normal oxygen conditions. Analysis showed miR-127/3p treatment significantly differed from the other two treatments. E) Cell migration results under hypoxic conditions. Analysis showed that miR-127/3p treatment significantly differed from the other two treatments. No significant difference was observed between hypoxia and normoxia.

## Description

MicroRNAs are short, non-coding nucleic acids, approximately 19-25 base pairs in length that regulate gene expression. MicroRNA expression analysis is becoming a newfound way to characterize cancer types (Lee and Dutta 2009) . There has been growing interest in determining the prognostic role of microRNAs in lung cancer (Wani, Majid et al. 2022) . Previous work in our lab found that increased levels of miR-127/3p in blood samples of breast cancer patients were associated with a favorable prognosis (Todorova, Byrum et al. 2022) . Further, UALCAN analysis showed lower levels of miR-127/3p in breast cancer when compared to healthy tissue. A Pan-Cancer analysis (fig. A) revealed that lower expression of miR-127/3p in cancerous tissue is present across various cancer types. Interestingly, upon UALCAN analysis, lung adenocarcinoma (LUAD) showed considerably lower levels of miR-127/3p when compared to other cancer types.

Lung cancer is one of the most common cancer types, and LUAD accounts for a large percentage of lung cancer cases. The general prognosis for LUAD is grim, and the average life expectancy for metastasized LUAD is 12.19 months (Zhao, Gao et al. 2023) . The most common sites of metastasis for LUAD are bone and other parts of the respiratory system (Riihimaki, Hemminki et al. 2014). These findings highlight the need for studies to understand the process of metastasis. To do this, our study uses wound healing (scratch) assays to measure the cell migration of lung cancer cells, a key feature of metastasized tumors (Liang, 2007) .

Recently, non-coding RNAs have been found to regulate cancer cell metastasis, and they are seen as potential biomarkers in the prognosis of lung cancer (Huang, Zhu et al. 2021) . Our study aims to measure the inhibitory effect of miR-127/3p on the migration of LUAD cells, an important first step in identifying miR-127/3p as a potential treatment and/or biomarker for lung cancer.

In other studies, miR-127/3p has been shown to inhibit the proliferation, migration, and invasion of triple-negative breast cancer cells by targeting various oncogenes associated with these mechanisms (Umeh-Garcia, Simion et al. 2020) . Further, miR-127/3p inhibits the migration and invasion of gastric cancer cells through a similar mechanism (Wang, Wang and Jiang 2019) .

To mimic the lung cancer tumor microenvironment, we included hypoxic incubation as part of our experimental setup. Reduced oxygen (hypoxia) in lung tissue is the most prominent feature of lung cancer tumors (Ziolkowska-Suchanek 2021). In as little as 30 minutes, hypoxia-induced gene expression by HIF1α leads to radiation resistance and overall aggression of cancer cells (Muz, de la Puente et al. 2015) .

As reported by others, 12- to 24-hour hypoxia incubations lead to increased necrosis and cell death in lung cancer cells. (Ancel, Perotin et al. 2021) To avoid this increased cell death and necrosis, we incubated LUAD cells for four hours under hypoxic conditions. Previous work in our lab showed changes in cell morphology and physiology after four-hour hypoxia incubations without noticeable changes in cell confluency (unpublished data). Further, our study focused on early changes in gene expression, making prolonged hypoxia treatment unnecessary.

Our study used wound healing (scratch) assays to mimic the cell migration of lung adenocarcinoma (A549) cells. We evaluated the effects of transfected miR-127/3p on cell migration under both normal oxygen and hypoxic conditions.

After transfection, plated cells were scratched, and cell migration was measured at 24, 48, and 72 hours. While common trends were observed throughout the experiment, only the 72-hour mark showed statistical differences between the controls and treated samples. Under normal oxygen conditions, miR-127/3p had inhibitory effects compared to lipofectamine only (p = 0.005) and the scrambled microRNA negative controls (p = 0.053). Cells transfected with miR-127/3p showed a 13% and 11% decrease in cell migration compared to the lipofectamine and scrambled miRNA control, respectively (fig. D).

While investigating the effects of miR-127/3p under hypoxic conditions, the results followed the same patterns. Transfecting A549 cells with miR-127/3p showed significantly different migration rates as compared to the negative control (p = 0.004), and a moderate difference was found when compared to the lipofectamine (p = 0.065). Transfection with miR-127/3p showed a 11.9% decrease from the negative control and a 7.5% decrease from the lipofectamine (fig. E). For all treatments, no significant difference was observed between hypoxic and normal oxygen conditions (p=0.365).

In conclusion, we found an inhibitory effect of miR-127/3p on the cell migration patterns of lung adenocarcinoma cells under both normal oxygen and hypoxic environments. These results help us understand the results previously mentioned, including the positive prognosis correlating with higher levels of miR-127/3p in the blood of breast cancer patients.

## Methods

This research was conducted as part of a course-based undergraduate research experience (CURE) at Ouachita Baptist University (Arkadelphia, Arkansas). All methods used for the project were “Cell Blocks” developed by the Cell Biology Education Consortium (CBEC). Cell Blocks are a series of modular written and video protocols. The flexibility of Cell Blocks allows researchers to design experiments that are of interest to them. For clarity, a link to the Cell Block used is included for each method.

**Cell Culture:** A549 cells (ATTC CRM-CCL-185) were grown in Dulbecco's Modified Eagle's Medium (DMEM), supplemented with 10% fetal bovine serum (FBS) and 2 mM L-glutamine for complete media. Unless otherwise noted, cells were grown in 5% CO _2_ at 37˚ C. CBEC Cell Growth A549 Cell Block: https://youtu.be/3Hg_LfHtY8M?si=z19lzHZcq7byi-IC

**Transfection with microRNA** : MiR-127/3p levels were increased in A549 cells by transfecting 50nM of the miR-127/3p mirVana ^™^ miRNA mimic (Invitrogen) into cells. The miR-127/3p mimic was transfected into cells displaying approximately 80% confluency using RNAi-MAX transfection reagent diluted in Opti-MEM ^™^ I Reduced Serum Medium (Gibco). As a negative control, 50nM of a negative (mirVana nonsense-scrambled) control was transfected into cells. Transfected cells were incubated under normoxic or hypoxic conditions for four hours. After incubation, cells were ‘scratched.’ After the scratch, the media was removed, and complete media was added to the cells. CBEC MicroRNA Transfection Cell Block: https://youtu.be/zT7Bd-SbNYY?si=0Mpg_8ki2sOe_ZJW

**Hypoxia** : Hypoxia was induced using a hypoxic incubation chamber (Stem Cell Technologies; catalog # 27310). The chamber was flooded for 4 min (20 PSI) with a mixed gas of 94.5 % N _2_ , 5% CO _2_ , and 0.5% O _2_ per the manufacturer's recommendations. Cells were incubated under hypoxia for 4 hours. After 4 hours of hypoxia treatment, cells were placed back into normal oxygen growth conditions. CBEC Hypoxia Cell Block: https://youtu.be/uHwonkoSdGo?si=_T8lMvoGZREtwh_H

**Scratch Assay** : After transfection and incubation under hypoxic or normal oxygen conditions, we scratched cell plates with a 200 μL pipette tip. For consistency in measurements, each scratch (bottom of the dish) was marked at two spots per well with a lab marker. Pictures of each marked spot were taken at 0, 24, 48, and 72 hours. To calibrate ImageJ for the measurements, a picture of a hemocytometer was taken at the same magnification. Using the known distance on the hemocytometer to set the scale, three measurements were taken for each picture (top, middle, and bottom), totaling six individual measurements for each scratch. These measurements were then averaged and used for further statistical analysis. CBEC Scratch Assay Cell Block: https://youtu.be/qbyUsSgIieU?si=FZjQzOgwOlM-S_pn

**Statistics** : Three biological replicates were conducted over six weeks. A minimum of two technical replicates were used each time. Normoxic and hypoxic treatments were conducted simultaneously, using cells from the same flask (passage number) for each biological replicate. Lipofectamine only and a microRNA non-sense (scrambled) sequence were used as negative controls. One-way analysis of variance (ANOVA) and post hoc Turkey’s HSD tests were used to compare cell migration among treatment groups for normal oxygen and hypoxia experiments using R (R Core Team 2024) .
